# Comparison of below-knee and above-knee amputations with demographic, comorbidity, and haematological parameters in patients who died

**DOI:** 10.1186/s13047-023-00635-x

**Published:** 2023-06-13

**Authors:** Cafer Özgür Hançerli, Necati Doğan

**Affiliations:** 1 Department of Orthopaedics and Traumatology, University of Health Sciences, Kanuni Sultan Süleyman Training and Research Hospital, Istanbul, Turkey; 2 Department of Orthopaedics and Traumatology, Başaksehir Çam and Sakura City Training and Research Hospital, Basaksehir Olimpiyat Bulvarı Yolu, 34480 Basaksehir, Istanbul, Turkey

**Keywords:** Above-knee amputation, Below-knee amputation, Diabetic foot, Mortality predictive parameters

## Abstract

**Background:**

This study aimed to establish mortality predictive parameters with a higher contribution to mortality by comparing the demographic data, comorbid factors, and haematological values of patients who underwent below-knee and above-knee amputation and had died during the follow-up period.

**Materials and methods:**

Between March 2014 and January 2022, 122 patients in a single centre who developed foot gangrene due to chronic diabetes and underwent below-knee or above-knee amputation were evaluated retrospectively. Patients who died of natural causes during the post-operative period were included in the study. Those who were amputated below the knee were assigned to Group 1, and those who were amputated above the knee were assigned to Group 2. The patients’ age, gender, side of amputation, comorbid diseases, American Society of Anaesthesiologists (ASA) score, Charlson comorbidity index (CCI), death time, and haematological values at the time of first admission were compared between the two groups and statistical analyses were performed.

**Results:**

Group 1 (*n* = 50) and Group 2 (*n* = 37) had similar distributions in terms of age, gender, side of operation, number of comorbidities, and CCI (*p* > 0.05). Group 2’s mean ASA score and c-reactive protein (CRP) levels were statistically higher than those of Group 1 (*p* < 0.05). Death time, albumin value, and HbA1c levels were statistically lower in Group 2 than in Group 1 (*p* < 0.05). There were no significant differences between the groups in haemogram, white blood cells (WBC), lymphocytes, neutrophils, creatinine, and Na values at the time of first admission (*p* > 0.05).

**Conclusion:**

A high ASA score, low albumin value, and high CRP value were significant predictors of high mortality. Creatinine levels and HbA1c values were quite ineffective in predicting mortality.

**Level of evidence:**

Level 3, retrospective comparative study.

## Introduction

The number of patients diagnosed with diabetes mellitus continues to climb due to the increasing population. In these patients, diabetes can result in outpatient gangrene, either alone or with the effect of comorbid diseases. While amputation is seen at least 5.8 percent in the general population, this rate is at least 46.1 percent in patients with diabetes. In addition, survival after amputation is quite short [1–3].

Amputation from the most distal level, where vascular flow and vitality are present, is preferred to minimize energy consumption and to make mobilization more convenient [4]. While debridement or minor amputation is applied to 74.8 percent of patients with diabetic foot, 20.3 percent need below-knee amputation and 6.9 percent need above-knee amputation [5, 6].

In the ongoing treatment of these chronic conditions, mortality is observed with the emergence of other organ failures, as well as amputation revisions. In the literature, we see that many haematological parameters and mortality indices have been tested, and predictive values have been revealed [7–10]. However, studies evaluating patients who died during convalescence are somewhat limited.

The aim of this study was to establish the mortality predictive parameters with a higher contribution to mortality by comparing the demographic data, comorbid factors, and haematological values of patients who underwent below-knee and above-knee amputation and died post-operatively.

## Materials and methods

### Study method and patient selection

Between March 2014 and January 2022, 122 patients who developed foot gangrene due to chronic diabetes and underwent below-knee or above-knee amputation in a single centre were evaluated retrospectively. Local ethics committee approval was obtained (KAEK/2021.01.3).

### Inclusion criteria

Patients with unilateral gangrene who were amputated directly below or above the knee, and who died before final follow-up were included in the study. Only natural deaths (as per the National Death Notification system) were included in the study.

### Exclusion criteria

Patients with a previous operation or revision history (except debridement) were excluded from the study. Bilateral amputations, patients still alive, and amputations due to acute embolism-atherosclerosis were excluded from the study, and deaths attributed to Covid-19 were excluded.

Those who were amputated below the knee were designated as Group 1, and those amputated above the knee were designated as Group 2.

Surgery preparations are started as soon as possible for all patients who are admitted to our clinic. To determine the levels at which the vascular structure is healthy, opinions are routinely requested from cardiovascular surgery (CVS). The most distal flow level is determined by CVS with Doppler ultrasound. In patients whose surgical preparations have been completed, the operation is started by the orthopaedist at the level specified by CVS, and if there is appropriate vitality, the level is maintained, but if muscle vitality and contractility are insufficient, it is increased to the upper level. Treatment of comorbidities after surgery are arranged by other clinicians, and patients are discharged to return for controls after blood sugar regulation is ensured. Compatible patients who do not develop wound problems during their outpatient follow-up period are mobilized with a prosthetic leg.

### Data collection

Age, gender, operation side, total number of comorbidities, ASA scores [11], CCI, and death time data of all patients were collected. Haemogram (HMG), WBC (white blood cell count), lymphocytes, neutrophils, albumin, creatinine, sodium (Na), HbA1c, and CRP values were collected in the first admission.

All operations were performed by surgeons following the same protocol using the same techniques and methods. All data were collected by the responsible surgeon.

### Statistical analysis

The IBM SPSS Statistics 26 (IBM, Chicago, IL, USA) program was used for statistical analysis. Explanatory statistics (mean, standard deviation, median, frequency, ratio, range) and data distribution were evaluated using the Shapiro–Wilk test. Student’s t-test was used to compare the data distributed between the two groups. A *p* value < 0.05 was established as the statistical significance level in all analyses.

## Results

The number of patients included in the study was 87. Group 1 included 50 patients, and Group 2 included 37 patients. No patient died during the perioperative period.

The mean age of Group 1 was 70.9 ± 6.9 (60–89), while the mean age of Group 2 was 74 ± 8.7 (65–98). The male/female ratio in Group 1 was 1.7, and 1.8 in Group 2. The groups had a statistically similar distribution in terms of age, gender, operation side, and number of comorbidities (*p* < 0.05) **(**Table 1).

**Table 1:** Demographic data of patients and key parameters

	**Below-knee amputations (Group 1) *N* = 50**	**Above-knee amputations (Group 2) *N* = 37**	***P* value**
**Age**	70,9 ± 6,9 (60–89)	74 ± 8,7 (65–98)	0,077
**Gender**	M:32 F:18	M:24 F:13	0,934
**Operation side**	R:24 L:26	R:17 L:20	0,851
**Total number of comorbidities**	2,52 ± 1,03 (1–5)	2,48 ± 1,04 (1–4)	0,882
**ASA scores**	ASA 2: 11	ASA 2: 2	
	ASA 3: 29	ASA 3: 25	
	ASA 4: 10	ASA 4:10	
**Mean ASA scores**	2,98 ± 0,65 (2–4)	3,21 ± 0,53 (2–4)	0,033*
**CCI**	6,2 ± 1,7 (3–10)	6 ± 1,4 (3–9)	0,413
**Death time (day)**	511,9 ± 558,2	309,6 ± 464,7	0,034*
	(3–1900)	(2–1875)	

^*^ *p* < 0.05 values are highlighted

While the mean ASA score was 2.9 in Group 1, it was 3.2 in Group 2. The average ASA score of Group 2 was statistically higher than that of Group 1 (*p* = 0.033). The mean CCI value in Group 1 was 6.2 ± 1.7 (3–10), while in Group 2 it was 6 ± 1.4 (3–9), and both groups had a similar distribution (*p* = 0.413). The mortality of Group 2 was higher when death times were compared (*p* = 0.034) (Table 1).

When the haematological parameters at the first admission were evaluated, there were no significant differences in terms of haemogram, WBC, lymphocytes, neutrophils, creatinine, and Na values between the groups (*p* > 0.05) (Table 2).

**Table 2:** Comparison of haematological parameters at the time of first admission

	**Below-knee amputations (Group 1) *N* = 50**	**Above-knee amputations (Group 2) *N* = 37**	***P* value**
Hmg	10 ± 2,3 (4,5–18,7)	9,5 ± 1,2 (6,3–12,25)	0,231
Wbc (count, thousand cells/mm3)	13,4 ± 7,6 (3,3–39,8)	14,1 ± 7,1 (2,9–39,6)	0,628
% lymphocytes	14,8 ± 7,9 (2,4–31,8)	12,2 ± 7,5 (2–39,8)	0,120
% neutrophils	75,4 ± 10,6 (50,6–92,2)	78,5 ± 11,7 (42,2–95,5)	0,195
Albumin	2,8 ± 0,5 (1,8–4,2)	2,6 ± 0,5 (1,5–4,5)	0,03*
Creatinine	1,6 ± 1,4 (0,3–6,9)	1,4 ± 1,2 (0,3–5,1)	0,488
Na	137,4 ± 4,9 (127–156)	137,1 ± 5,6 (128–153)	0,843
HbA1c	7,4 ± 1,5 (5–12,6)	6,7 ± 1,5 (5–11,3)	0,018*
Crp	105,6 ± 91,3 (3–361)	167,7 ± 107,2 (24–415)	0,002*

^*^ *p* < 0.05 values are highlighted

The mean albumin value of Group 2 was lower than that of Group 1 (*p* = 0.03). The mean CRP value was higher in Group 2 (*p* = 0.02). The mean HbA1c value was lower in Group 2 (*p* = 0.018) (Table 2).

## Discussion

We found that above-knee amputations had a shorter survival than below-knee amputations. In line with this data, all available data of both groups were compared, and data with significant statistical differences were evaluated as having a higher or lower contribution to mortality. When we compared the demographic, comorbid, and haematological values of both groups, it was revealed that age, gender, side, number of comorbidities, and CCI and HMG, WBC, lymphocytes, neutrophils, creatinine, and Na values at the time of first admission did not make a significant predictive difference in the mortality criterion. It was revealed that a high ASA score, low albumin level at first admission, and high CRP levels resulted in higher mortality. Although the HbA1c value was low, its occurrence in the group with high mortality meant that it was not associated with mortality.

Some publications state that older age and male gender are associated with higher mortality [7, 8]. In our study, the mean age of the patients was 70.9 in Group 1, 74 in Group 2, and male gender was dominant in both groups. Although the age and gender distributions of both groups were similar, Group 2 revealed higher mortality.

While there are publications stating that mortality increases as the number of comorbid diseases increases [9, 10], our study also revealed that although the number of comorbidities was equal in the two groups, those who underwent above-knee amputation had a higher probability of mortality. Although mortality was high in both groups, the reason Group 2’s was higher may be related to the stage of the disease rather than the number of comorbidities.

The ASA score is a parameter that indicates the perioperative anaesthesia risk of the patients, and the hospital routinely evaluates patients’ cardiological (echocardiogram), pulmonary (respiratory function test and blood gas), and internal medicine (liver and kidney function, electrolyte, and hormone) status and gives scores in a transparent way. We have revealed that an increase in this score is an important factor that determines the patient’s survival. In the literature, we see that the survival relationship with ASA is also established in other diseases [11]. Considering the type of anaesthesia, we see that as the ASA score increases, the preference for general anaesthesia over regional anaesthesia increases. We believe that general anaesthesia is preferred for the stabilization of patients who are likely to need intensive care after the operation.

If the CCI is > 5, the mortality risk is stated as 22.3 percent in some publications [10]. While the CCI was 6.2 in Group 1, it had a mean of 6 in Group 2. Group 1 resulted in a mortality of approximately 2 years on average, and Group 2 with an average mortality of approximately 1 year. It can be concluded that a high mortality rate of 5 or more is predicted, but although both groups were similar in our study, the higher mortality rate of Group 2 alone could not constitute a high predictive factor.

Haemogram, WBC, lymphocytes, neutrophils, Na, and creatinine were similar in the two groups, suggesting that they are not predictors of high mortality. Although a low haemogram level has also been shown as a mortality indicator [10], it did not make a significant difference in our study. However, the mean haemogram values in both groups (Group 1, HMG 10; Group 2, HMG 9.5) were below normal values. Many publications have suggested that high creatinine levels and dialysis dependence are associated with higher mortality [7–10]. In our study, while the mean creatinine level in Group 1 was 1.6, it was 1.4 in Group 2; 15 patients in Group 1 and 5 in Group 2 were receiving dialysis. It seems unwarranted to suggest that creatinine levels have an effect on mortality. Although we cannot claim that it did not contribute to mortality, as our CCI results were similar in both groups, we can only say that it is not the main factor that influences mortality.

According to our study, a low albumin value causes greater mortality. If we consider low albumin as a sign of liver failure, it can be argued that liver problems cause higher mortality than kidney disease (high creatinine levels). There are publications in the literature supporting the idea that low albumin increases mortality [12, 13]. In addition, when low albumin accompanies high CRP, it can be an indicator of infection [14]. Low albumin level may also be associated with septicemia.

We have seen that a high level of CRP also causes higher mortality. It can be easily believed that the extent of septicaemia in the process and the strength of the patient’s immune system affect mortality. In the literature, it has been stated that resistant bacteria cause higher mortality [9] and that CRP may also be a predictor of high mortality [12]. The aetiology of a high CRP can be evaluated as septicaemia.

Although the HbA1c level was low in Group 2, the high mortality rate indicates that systemic organ damage caused by chronic glucose elevation is more important than HbA1c. However, although there are publications in the literature [15, 16] stating that high HbA1c increases mortality, it is a parameter that can be improved with good treatment and diet.

In other mortality predictive studies in the literature, parameters such as functional status, steroid use, delirium, thrombocythemia, insulin use, and heart diseases have been evaluated, and they report that these parameters also contributed to mortality [7–10]. We did not evaluate these parameters in our study, but we consider them to be parameters that can be stabilized, since we have shown the necessary care to obtain support from related branches for the stabilization of additional diseases.

There are some limitations in our study. First, the study has limitations due to its retrospective nature and having been conducted in a single centre, a lack of power analysis, inadequacy in randomization, and not using blinding techniques. It is not known how much regular care some neurological, personal, and chronic diseases receive in the postoperative period. Standardizing the post-discharge stability of patients’ chronic diseases will yield clearer results. Again, these patients were eligible for inclusion by confirming their haematological evaluations at certain periods. Multicentre prospective studies are needed for high-evidence findings.

## Conclusions

Although advanced age, male gender, multiple co-morbidities, high ASA score, high CCI value, low haemogram value, low albumin value, and high CRP can be associated with mortality in diabetic gangrene-induced amputations, only high ASA scores, low albumin values, and high CRP values were found to be significant predictors of high mortality. Creatinine levels and HbA1c values were quite irrelevant in predicting mortality.

## Data Availability

The datasets used and/or analysed during the current study are available from the corresponding author on reasonable request.
